# An epidemiological study of pediatric COVID-19 in the era of the variant of concern

**DOI:** 10.1371/journal.pone.0267035

**Published:** 2022-04-15

**Authors:** Chanapai Chaiyakulsil, Paskorn Sritipsukho, Araya Satdhabudha, Pornumpa Bunjoungmanee, Auchara Tangsathapornpong, Phakatip Sinlapamongkolkul, Naiyana Sritipsukho

**Affiliations:** 1 Faculty of Medicine, Division of Pediatric Critical Care, Department of Pediatrics, Thammasat University Hospital, Thammasat University, Pathumthani, Thailand; 2 Faculty of Medicine, Division of Pediatric Allergy and Immunology, Department of Pediatrics, Thammasat University, Pathumthani, Thailand; 3 Center of Excellence in Applied Epidemiology, Thammasat University, Pathumthani, Thailand; 4 Faculty of Medicine, Division of Pediatric Pulmonology, Department of Pediatrics, Thammasat University, Pathumthani, Thailand; 5 Faculty of Medicine, Division of Pediatric Infectious Disease, Department of Pediatrics, Thammasat University, Pathumthani, Thailand; 6 Faculty of Medicine, Division of Pediatric Hematology and Oncology, Department of Pediatrics, Thammasat University, Pathumthani, Thailand; 7 Thammasat Postdoctoral Fellowship, Thammasat University, Pathumthani, Thailand; Auburn University, UNITED STATES

## Abstract

**Background:**

There were limited epidemiological data of pediatric COVID-19 in Asia outside China, especially during the era of the variants of concern such as the Delta and Alpha variants. The objective was to describe the clinical epidemiology of pediatric COVID-19 in a tertiary care center in Thailand from April to August 2021. The identification of risk factors for the development of pneumonia in these children was also performed.

**Methods:**

This retrospective cohort study was conducted by retrospective chart review of all children aged 0–15 years admitted to Thammasat University Hospital care system during the study period. The risk factors for the development of pneumonia were analyzed using logistic regression.

**Results:**

A total of 698 children were included for analysis, of which 52% were male. The mean age of the cohort was 7.2 + 4.5 years old. Radiographic pneumonia was identified in 100 children (14.3%) and a total of 16 children (2.3%) were diagnosed with severe and critical diseases. The mortality rate was 0.1%. Children younger than 1 year and children with comorbidity were at higher risk of developing pneumonia (Adjusted odds ratios 2.99 (95% confidence interval (CI): 1.56–5.74) and 2.32 (95% CI: 1.15–4.67), respectively).

**Conclusion:**

In the era of the variants of concern, the proportion of children with severe and critical diseases remained low. However, prudence must be taken in caring for younger children and children with comorbidity.

## Introduction

A novel severe acute respiratory syndrome coronavirus 2 (SARS-CoV-2) infection played a major role in causing a global pandemic and healthcare catastrophe. The Coronavirus disease 2019 (COVID-19), caused by SARS-CoV2, was first reported in humans in December 2019 in Wuhan, China [1–3]. According to the World Health Organization (WHO), at the time of the writing of this manuscript, more than 220 million people were infected with COVID-19 worldwide with a shocking number of more than 4 million deaths [4]. Since its emergence, it had been found that the infection rates in children were disproportionately lower than adults and children tend to have a less severe clinical course [5].

The first large epidemiological cohort study of children infected with COVID-19 was first published by the Chinese Center of Disease Control (CDC) which demonstrated a low incidence of severe and critical illness in children (5.9%; N = 2135) and only one death [3]. Currently, there were only 3 large meta-analyses and systematic reviews in children with COVID-19. A systematic review of 7,780 children with COVID-19 infection, which encompassed a total of 131 studies, revealed only 1% of children with critical illness and a 0.1% mortality rate [6]. Another meta-analysis, comprising of 48 studies, also demonstrated that only approximately 12% of children had a severe and critical illness. This meta-analysis revealed no mortality [7]. A large meta-analysis illustrated that 5.1% of children with comorbidities and 0.2% of children without comorbidities were found to have severe COVID-19 [8].

Interestingly, the studies included in these meta-analyses were conducted mostly in China, the United States, and Europe with small data from Singapore. Only one cohort study with a small population (N = 260) described the clinical characteristics of children with COVID-19 in Singapore, Japan, and other south Asian countries.^5^ Thus, the epidemiological data of children with COVID-19 in Southeast Asia remains lacking. Furthermore, most studies were conducted in 2020, where several variants of concern (VOC), such as B.1.1.7 (Alpha), B.1.351 (Beta), and B.1.617.2 (Delta) variants, were not presented. VOC was defined by the US CDC as the variant for which there was evidence of increasing transmissibility, higher severity, reduction of neutralizing antibodies after vaccination, and reduced effectiveness of treatment modalities [9]. Therefore, there was a major lack of epidemiological data of children infected with COVID-19 in 2021.

The VOC of the Alpha variant was first reported in Thailand in January 2021 and started surging in April. The Delta variant which was first reported in June further added the scourge throughout the country. The primary objective of this study was to describe the clinical epidemiology of pediatric COVID-19 in a tertiary care center in Thailand from April to August 2021. At Thammasat University Hospital (TUH), we admitted all children who were diagnosed with COVID-19 into either the hospital, field hospital, or the home isolation program based on the risk factors and the severity of the patients. Thus, the secondary objective was to compare the clinical characteristics, severity, and outcomes of children admitted to these different facilities. The identification of risk factors for the development of pneumonia in these children was also performed.

## Materials and methods

### Study design

This retrospective cohort study was conducted by retrospective chart review. The Human Research Ethics Committee of the Faculty of Medicine, Thammasat University approved this study (EC number: MTU-EC-PE-0-222/64). The committee is in full compliance with the Declaration of Helsinki, The Belmont Report, CIOMS guidelines, and the International Practice. Due to the study’s retrospective nature, informed consent and assent were waived.

### Participants

All children from age 0–15 years old who were admitted with COVID-19 to Thammasat University Hospital, field hospital, and home isolation program were included for the analysis. All children were diagnosed using confirmatory tests of reverse transcriptase-polymerase chain reaction (RT-PCR) or antigen test kit (ATK) for COVID-19. RT-PCR was a routine test required for admission into the system before August 2021 due to the unavailability of ATK throughout the country. At the start of August, ATK was available for the general public and could be used for admission into the home isolation system in an attempt to reduce spread within the community. Nevertheless, as per the hospital policy, all patients who were admitted to the main hospital and the field hospital required RT-PCR for admission throughout the study period.

TUH is a large tertiary care, university hospital with approximately 100 pediatric beds and 6-beds mixed medical and surgical pediatric intensive care unit. As part of the Thailand healthcare system and the attempt to contain the spread of infection, our policy was to admit all the patients who were diagnosed with COVID-19 into the system. The decision for the admission site was based on the severity of symptoms in each patient. Children with improving clinical symptoms would be transferred to the field hospital or home isolation program whilst children with deteriorating symptoms would be referred back to the main hospital.

### Operational admission site aside from the main hospital

#### Field hospital

As the numbers of infected individuals were growing throughout the country in April 2021, TUH started a field hospital service. The staff dormitory of Thammasat University was transformed into a 470-beds facility for admittance of the patients who were diagnosed with mild to moderate COVID-19 infection. All admitted patients received daily telephone follow-up by medical personnel and medications, including antivirals, were prescribed according to the national guidelines. All patients received a chest roentgenogram on admission. In adults, an oxygen supplementation via oxygen cannula lower than 5 liters were acceptable to stay in the field hospital without having to transfer to the main hospital. In children, any patients requiring oxygen supplementation were referred back to the main hospital.

#### Home isolation program

With the emergence of the Delta variant in June, TUH initiated a home isolation program to care for asymptomatic patients or patients with mild symptoms who could perform self-quarantine at home. All patients received daily sustenance and daily telephone follow-up by both doctors and nurses. Patients were required to perform the self-check on body temperature and oxygen saturation at least twice per day and report the findings to the medical personnel. Supportive medications, as well as antivirals, were prescribed according to the indications and the national guidelines. Patients with worsening symptoms would be transferred to the main hospital. To prevent the spread of infection to the community during the transport, the roentgenogram of the chest was limited only to those with clinical signs suggestive of pneumonia.

### Data collection

All demographic data, laboratory findings (when done), severity, treatments, and outcomes in terms of mortality of each child were reviewed and recorded. The definitions of severity were defined according to the WHO as described in Table 1 [10]. Children with incomplete data in terms of severity and clinical outcomes were excluded from the analysis. All roentgenogram of the chest was reviewed and dictated by the attending radiologists. The diagnosis of pneumonia was made by either clinical signs of lower respiratory tract infection such as dyspnea, chest pain, or shortness of breath or the abnormal radiographic findings dictated by the radiologists. Abnormal radiographic findings compatible with pneumonia included ground-glass opacity, alveolar, or interstitial infiltration.

**Table 1:** A severity level for patients with COVID-19 infection; Adapted from reference no 10.

Severity level	Signs and symptoms
**1. Asymptomatic**	Asymptomatic
**2. Mild COVID-19**	Signs and symptoms of respiratory tract infection or gastrointestinal symptoms without pneumonia
**3. Moderate COVID-19**	Pneumonia that did not fulfill the criteria of severe COVID-19
**4. Severe COVID-19**	Pneumonia with oxygen saturation at room air < 90%* Tachypnea > 30 per minute in adults or children > 5 years old > 60 per minute in children < 2 months > 50 per minute in children 2–11 months > 40 per minute in children 1–5 years old **Severe respiratory distress**
**4. Critical COVID-19**	Requiring mechanical ventilation, inotropes, or vasopressors Fulfilled the criteria for acute respiratory distress syndrome or septic shock

*Caution: An oxygenation saturation of 90–94% at room air might be an early sign for patient deterioration. These patients were considered to have severe disease if requiring respiratory support.

### Statistical analyses

All demographic data, laboratory findings, severity, treatments, and outcomes were analyzed using descriptive statistics. The severity and outcomes were compared among different months using Chi-square and ANOVA as appropriate. The risk factors for the development of pneumonia were analyzed using univariate and multivariate logistic regression. The statistically significant variables from the univariate logistic regression were further analyzed in the multivariate logistic regression to find the risk factors for the development of pneumonia. All statistical analyses were performed using SPSS Version 24 (IBM Corporation, Armonk, New York).

## Results

### Demographic data

A total of 698 children were admitted to the TUH care facilities during the study period. No children were excluded from the analysis. No patient within this cohort had an incidental finding of a positive result for other reasons such as surgery or trauma. The diagnoses were made by RT-PCR in 92.4% of the cohort, whilst the remaining were made using ATK. Approximately 52% were male. The mean age of the cohort was 7.2 + 4.5 years old. One-hundred and fifty-four children (22.1%) were found to be overweight and obese (weight for height > + 2 standard deviation (SD)).

Underlying diseases were found in 54 children (7.8%). Allergic and pulmonary comorbidities constituted the majority of the co-morbidities of the children (28/54 children; 51.8%). The most commonly reported comorbidities were allergic rhinitis (14/54 children; 25.9%) and asthma (10/54; 18.5%). No patient with chronic lung disease was found within this cohort. Four children had congenital heart diseases, three had a ventricular septal defect and one patient had an atrioventricular canal defect. Hematologic co-morbidities included 2 cases of hematologic malignancies, 3 cases of thalassemia, 2 cases of glucose-6-phosphate dehydrogenase deficiency, and one case of idiopathic thrombocytopenia. Three patients had cerebral palsy requiring total assistance. Developmental comorbidities were autism and attention deficit disorder, which only require little or no support during daily living.

The majority of patients (52.1%) reported having respiratory symptoms and 4.1% reported mixed respiratory and gastrointestinal symptoms. Approximately 35.9% of the children reported no clinical symptoms. Isolated fever was found in 2.6% of children.

During the study period, 140 children were admitted to the hospital, 296 to the field hospital, and 262 to the home isolation program. Three patients from the home isolation program and one patient from the field hospital were transferred to the hospital due to poor intake and mild dehydration and were included in the hospital cohort. There was a significantly higher proportion of children younger than 1-year-old admitted to the hospital compared to the field hospital and the home isolation program (17% VS 6.4% VS 3.4%, respectively; *p* < 0.001). Mean age, body mass index, nutritional status, and underlying comorbidities were not significantly different among the admission site. The demographic data based on the site of admission were described in Table 2.

**Table 2:** Demographic data based on site of admission.

	Population (N = 698)	Hospital (N = 140)	Field Hospital (N = 296)	Home isolation program (N = 262)
**Mean age (Years + SD)** **Age < 1 year old (N; %)** * Gender (N; %) Male Female **Mean BMI (kg/meter**^**2**^ **+ SD)** **Nutritional status (N; %)** • Wasting (W/H below -2 SD) • Thin (W/H between -1.5 SD and– 2 SD) • Normal (W/H between + 2 SD to– 1.5 SD) • Overweight (W/H +2 SD to + 3SD) • Obese (W/H > + 3 SD)	7.2 + 4.5 52 (7.4) 363 (52.0) 335 (48.0) 18.7 + 5.5 60 (8.6) 32 (4.6) 452 (64.7) 64 (9.2) 90 (12.9)	6.2 + 4.7 24 (17.0) 74 (52.9) 66 (47.1) 18.8 + 5.9 9 (6.4) 7 (5.0) 96 (68.6) 14 (10.0) 14 (10.0)	7.5 + 4.4 19 (6.4) 159 (53.7) 137 (46.3) 18.9 + 5.8 25 (8.5) 14 (4.7) 181 (61.1) 30 (10.2) 46 (15.5)	7.3 + 4.4 9 (3.4) 130 (49.6) 132 (50.4) 18.5 + 4.8 26 (9.9) 11 (4.2) 175 (66.8) 20 (7.6) 30 (11.5)
**Underlying diseases (N; %)** • None • Allergy • Pulmonology • Hematology and Oncology • Cardiology • Neurology • Developmental • Dermatology • Gastrointestinal • Endocrine • Nephrology • Preterm	644 (92.2) 17 (2.4) 11 (1.6) 8 (1.3) 4 (0.6) 4 (0.6) 4 (0.6) 2 (0.3) 1 (0.1) 1 (0.1) 1 (0.1) 1 (0.1)	123 (87.8) - 3 (2.2) 3 (2.2) 3 (2.2) 2 (1.4) 1 (0.7) 1 (0.7) 1 (0.7) 1 (0.7) 1 (0.7) 1 (0.7)	275 (92.9) 11 (3.8) 3 (1.0) 3 (1.0) 1 (0.3) 1 (0.3) 2 (0.7) - - - - -	246 (93.9) 6 (2.3) 5 (1.8) 2 (0.8) - 1 (0.4) 1 (0.4) 1 (0.4) - - - -
**Symptoms (N; %)** * • Respiratory • None • Mixed respiratory and gastrointestinal • Isolated fever • Gastrointestinal • Mixed respiratory and dermatological • Neurological • Dermatological • Mixed gastrointestinal and dermatological	364 (52.1) 251 (35.9) 29 (4.1) 18 (2.6) 16 (2.3) 14 (2.0) 3 (0.4) 2 (0.3) 1 (0.1)	93 (66.4) 29 (20.6) 7 (5.0) 2 (1.5) 4 (2.8) 2 (1.5) 2 (1.5) 1 (0.7) -	119 (40.2) 146 (49.4) 6 (2.0) 10 (3.4) 5 (1.7) 7 (2.4) 1 (0.3) 1 (0.3) 1 (0.3)	152 (58.0) 76 (29.0) 16 (6.1) 6 (2.3) 7 (2.7) 5 (1.9) - - -
**Confirmatory test (N; %)** * • RT-PCR • Antigen test kit	645 (92.4) 53 (7.6)	140 (100.0) -	296 (100.0) -	209 (79.8) 53 (20.2)
Severity (N; %) * • Asymptomatic • Mild • Moderate • Severe • Critical	223 (31.9) 374 (53.6) 85 (12.2) 12 (1.7) 4 (0.6)	21 (15.0) 66 (47.1) 37 (26.4) 12 (8.6) 4 (2.9)	128 (43.2) 133 (45.0) 35 (11.8) - -	74 (28.2) 175 (66.8) 13 (5.0) - -
Chest radiographic findings (N; %) * • Normal • Abnormal • Not done	355 (50.8) 100 (14.4) 243 (34.8)	88 (62.9) 52 (37.1) -	261 (88.2) 35 (11.8) -	6 (2.3) 13 (5.0) 243 (92.7)
Respiratory support (N; %) * • None • Oxygen cannula • Oxygen box • High flow nasal cannula • Mechanical ventilator Mean duration of ventilator support (Days + SD) *	682 (97.7) 7 (1.0) 1 (0.1) 4 (0.6) 4 (0.6) 11.2 + 7.1	124 (88.5) 7 (5.0) 1 (0.7) 4 (2.9) 4 (2.9) 11.2 + 7.1	296 (100.0) - - - - -	262 (100.0) - - - - -
Antiviral treatment (N; %) * • Favipiravir • Remdesivir **Mean duration of antiviral (days + SD)** Antibiotics treatment (N; %) * Mean duration of antibiotics (days + SD) * Steroid treatment (N; %) * Mean duration of steroids (days; SD) *	117 (16.8) 2 (0.3) 5.3 + 1.4 19 (2.7) 8.0 + 5.0 11 (1.6) 7.0 + 1.3	45 (32.1) 2 (1.4) 5.9 + 2.1 19 (13.6) 8.1 + 5.0 11 (7.8) 7.0 + 1.3	31 (10.5) - 5.0 + 0.0 0 (0.0) - 0 (0.0) -	41 (15.6) - 5.0 + 0.0 0 (0.0) - 0 (0.0) -

*P < 0.05.

SD = standard deviation; RT-PCR–reverse transcriptase polymerase chain reaction; BMI = body mass index; W/H = Weight for height.

The number of patients being admitted to the TUH care facilities increased consistently with the surge of patients throughout the country with 46 patients in April to 266 patients in August. In April, approximately 52.2% reported no clinical symptoms whilst in August only 21.4% of the patients reported no symptoms. The proportion of the reported symptoms was significantly different among the months during the study period and admission site (*p* < 0.001). Mean age, gender, body mass index, nutritional status, underlying comorbidities, and mean duration of hospital stays were also not significantly different among months of admission. The demographic data of the patients based on months of admission were summarized in Table 3.

**Table 3:** Demographic data based on month.

	April (N = 46)	May (N = 65)	June (N = 125)	July (N = 196)	August (N = 266)
**Symptoms (N; %)** * • Respiratory • None • Mixed respiratory and gastrointestinal • Isolated fever • Gastrointestinal • Mixed respiratory and dermatological • Neurological • Dermatological • Mixed gastrointestinal and dermatological	20 (43.4) 24 (52.2) - - - 1 (2.2) - 1 (2.2) -	25 (38.5) 36 (55.4) 1 (1.5) - 2 (3.1) 1 (1.5) - - -	52 (41.6) 66 (52.8) 3 (2.4) 2 (1.6) 2 (1.6) - - - -	102 (52.0) 68 (34.7) 4 (2.0) 7 (3.7) 8 (4.1) 4 (2.0) 2 (1.0) - 1 (0.5)	165 (62.0) 57 (21.4) 21 (7.9) 9 (3.4) 4 (1.5) 8 (3.0) 1 (0.4) 1 (0.4) -
**Confirmatory test (N; %)** * • RT-PCR • Antigen test kit	46 (100.0) -	65 (100.0) -	125 (100.0) -	196 (100.0) -	213 (80.1) 53 (19.9)
**Severity (N; %)** * • Asymptomatic • Mild • Moderate • Severe • Critical	20 (43.5) 16 (34.8) 9 (19.6) 1 (2.1) -	35 (53.9) 22 (33.8) 6 (9.2) 2 (3.1) -	53 (42.4) 42 (33.6) 28 (22.4) 2 (1.6) -	58 (29.6) 105 (53.6) 25 (12.8) 6 (3.0) 2 (1.0)	57 (21.4) 189 (71.1) 17 (6.4) 1 (0.4) 2 (0.8)
**Chest radiographic findings (N; %)** * • Normal • Abnormal • Not done	36 (78.3) 10 (21.7) -	58 (89.2) 7 (10.8) -	95 (76.0) 30 (24.0) -	94 (49.0) 33 (16.8) 69 (35.2)	72 (27.1) 20 (7.5) 174 (66.4)
Admission (N; %) * • Hospital • Field hospital • Home isolation **Respiratory support (N; %)** • None • Oxygen cannula • Oxygen box • High flow nasal cannula • Mechanical ventilator	22 (47.8) 24 (52.2) - 45 (97.8) 1 (2.2) - - -	21 (32.3) 44 (67.7) - 63 (97.0) - 1 (1.5) 1 (1.5) -	51 (40.8) 74 (59.2) - 123 (98.4) 2 (1.6) - - -	30 (15.3) 87 (44.4) 79 (40.3) 188 (96.0) 3 (1.5) - 3 (1.5) 2 (1.0)	16 (6.0) 67 (25.2) 183 (68.8) 263 (98.8) 1 (0.4) - - 2 (0.8)
**Antiviral treatment (N; %)** * • Favipiravir • Remdesivir **Antibiotics treatment (N; %)** **Steroid treatment (N; %)** *	3 (6.5) - 1 (2.2) 0 (0.0)	8 (12.3) - 2 (3.1) 2 (3.1)	22 (17.6) - 1 (0.8) 0 (0.0)	24 (12.3) 1 (0.5) 10 (5.1) 6 (3.1)	59 (22.2) 1 (0.4) 5 (1.9) 3 (0.8)

*P < 0.05.

BMI = body mass index.

### Disease severity

A total of 223 children (31.9%) were classified as having asymptomatic infection by the WHO criteria, 374 children (53.6%) with a mild infection, 85 children (12.2%) with moderate infection, and the remaining 16 children (2.3%) with severe and critical infection. Seven children with severe and critical infections had underlying comorbidities, whilst the remaining 9 children had no comorbidities. Sixteen patients (2.3%) required respiratory support; seven patients required oxygen cannula, 1 patient required oxygen box, 4 patients were on high flow nasal cannula support, and 4 patients were mechanically ventilated. One mortality (0.1%) was found in this cohort and was initially admitted to the main hospital. The patient was a preterm infant with low birth weight who suffered from acute respiratory distress syndrome with persistent pneumothorax. The mean duration of ventilator support for the whole cohort was 11.2 + 7.1 days. The severity was higher among patients admitted to the hospital when compared to those admitted to the field hospital and home isolation program (*p* < 0.001). Six patients (0.8%) required intensive care unit admission and 3 patients received inotropic support. The mean duration of intensive care unit admission was 10.5 + 7.9 days. Respiratory support utilization was not significantly different among months during the study period (*p =* 0.142).

Antiviral medications were prescribed in 120 patients (17.1%). Favipiravir was served as the major antiviral medication in Thailand and Remdesivir was reserved for severe and critical infection. Only two patients received Remdesivir during the study period due to the medication unavailability. The mean duration of antiviral use was 5.3 + 1.4 days. The rate of antiviral prescription was significantly higher in the hospital group when compared to the field hospital and the home isolation group (33.5% VS 10.5% VS 15.6%, respectively; *p* < 0.001). The proportion of patients receiving antiviral therapy was significantly different among months with the highest rate of prescription of 22.2% in August (*p* = 0.008). The majority of febrile patients had a subsiding fever within 48 hours after receiving antiviral therapy. Antibiotics were co-administered in 19 patients (2.7%) with a mean duration of 8.0 + 5.0 days. One patient had a positive hemoculture for *Klebsiella Pneumoniae* after five days of hospital admission. Steroid was given in 11 patients (1.6%) with a mean duration of 7.0 + 1.3 days. No antibiotics and steroids were administered in the field hospital and the home isolation patients.

With the increasing numbers of admitted patients from April to August, the disease severity was statistically different among months during the study period (*p* < 0.001). The decline in asymptomatic patients was observed in July and August with increasing numbers of patients with mild symptoms. Critical patients were also observed only in July and August. Steroid use was only observed in May, July, and August with the majority of utilization in July and August. The proportion of children receiving antibiotics was not significantly different among months (*p =* 0.151). The data on severity, respiratory support, antiviral, antibiotics, and steroids used were described in Tables 2 and 3.

### Pneumonia and associating factors

A total of 455 patients (65.1%) received a chest roentgenogram. All patients admitted to the hospital and the field hospital received at least one roentgenogram. However, only 19 out of 262 children (13.7%) in the home isolation program received the chest roentgenogram due to clinical suspicion of pneumonia. Radiographic pneumonia was observed in 100 patients (14.3% for the whole cohort and 22.0% among those receiving chest roentgenogram). All patients who exhibited signs of lower respiratory tract infection had radiographic pneumonia. The proportion of patients with pneumonia was significantly higher in the hospital when compared to the field hospital and the home isolation program (52% VS 35% VS 13%; *p* < 0.001). The majority of patients (69%) with pneumonia exhibited respiratory symptoms. Interestingly, 28 patients (28%) with radiographic pneumonia reported no clinical symptoms. None of these patients required respiratory support.

The mean age of the patients was not significantly different among groups (p = 0.337). There was a significantly greater proportion of children younger than 1-year-old in the pneumonia group when compared to the patients without pneumonia (19.0% VS 6.7%; *p* < 0.001). Children with pneumonia had significantly higher body mass index than the children without pneumonia (19.5 + 6.4 kg/m^2^ VS 18.7 + 5.6; *p* < 0.001). The nutritional status was not significantly different among groups (*p =* 0.368). Twenty-four children with pneumonia (24%) were found to be either overweight or obese (weight for height > + 2 standard deviation), which did not demonstrate a significantly different proportion when compared to those without pneumonia (*p =* 0.851). There were a significantly higher proportion of patients with underlying comorbidities among the patients with pneumonia (*p =* 0.009). Twenty-three patients (6.5%) in the non-pneumonia group and 63 patients (63%) in the pneumonia group received antiviral medication (*p* < 0.001). Five patients (1.4%) in the non-pneumonia group and 14 patients (14.0%) in the pneumonia group received antibiotics treatment (*p* < 0.001). No children in the non-pneumonia group received steroid therapy. The mean duration of hospital stays, antibiotics, and antiviral medications were not significantly different among groups.

In terms of laboratory investigations, the complete blood count was performed in 282 patients (40.4%), 218 patients (61.4%) in the non-pneumonia group, and 64 (64.0%) in the pneumonia group. Children with pneumonia had a significantly lower mean hematocrit (p = 0.047), higher mean white blood cells count (WBC) (*p* < 0.001), higher mean absolute neutrophils count (ANC) (*p* < 0.001), and higher mean absolute lymphocytes count (ALC) (*p* < 0.001). There was also a significantly higher proportion of children having leukocytosis (white blood cell count > 11,000 cells/cumm^3^) in the pneumonia group (*p* < 0.001). The data comparing patients with and without pneumonia were demonstrated in Table 4.

**Table 4:** Demographic data based on diagnosis of radiographic pneumonia.

	Without Pneumonia (N = 355)	Pneumonia (N = 100)	P-value
**Mean age (Years + SD)** **Age group (N; %)** * Age < 1 year old Age 1–5 years old Age 5–10 years old Age > 10 years old **Gender (N; %)** Male Female **Mean BMI (kg/m**^**2**^ **+ SD)** * Nutritional status (N; %) • Wasting (W/H below -2 SD) • Thin (W/H between -1.5 SD and– 2 SD) • Normal (W/H between + 2 SD to– 1.5 SD) • Overweight (W/H +2 SD to + 3SD) • Obese (W/H > + 3 SD)	7.5 + 4.3 24 (6.7) 101 (28.5) 120 (33.8) 110 (31.0) 192 (54.1) 163 (45.9) 18.7 + 5.6 29 (8.2) 20 (5.6) 223 (62.8) 34 (9.6) 49 (13.8)	5.9 + 5.0 19 (19.0) 37 (37.0) 16 (16.0) 28 (28.0) 46 (46.0) 54 (54.0) 19.5 + 6.4 7 (7.0) 1 (1.0) 68 (68.0) 11 (11.0) 13 (13.0)	0.337 < 0.001 0.153 0.043 0.368
**Underlying diseases (N; %)** * • None • Allergy • Pulmonology • Hematology and Oncology • Cardiology • Neurology • Developmental • Dermatology • Gastrointestinal • Endocrine • Nephrology • Preterm	331 (93.2) 10 (2.8) 5 (1.4) 4 (1.1) 2 (0.6) 1 (0.3) 2 (0.6) - - - - -	85 (85.0) 1 (1.0) 2 (2.0) 2 (2.0) 2 (2.0) 2 (2.0) 1 (1.0) 1 (1.0) 1 (1.0) 1 (1.0) 1 (1.0) 1 (1.0)	0.009
**Symptoms (N; %)** * • Respiratory • None • Isolated fever • Mixed respiratory and gastrointestinal • Mixed respiratory and dermatological • Gastrointestinal • Neurological • Dermatological • Mixed gastrointestinal and dermatological	161 (45.4) 152 (42.8) 12 (3.4) 9 (2.5) 9 (2.5) 6 (1.7) 3 (0.8) 2 (0.6) 1 (0.3)	59 (59.0) 28 (28.0) - 9 (9.0) 1 (1.0) 3 (3.0) - - -	0.005
**Admission (N; %)** * • Hospital • Field hospital • Home isolation **Mean hospital stay (days + SD)**	88 (24.8) 261 (73.5) 6 (1.7) 4.0 + 2.6	52 (52.0) 35 (35.0) 13 (13.0) 5.9 + 7.1	<0.001 0.423
**Antiviral treatment (N; %)** **Mean duration of antiviral (days + SD)** **Antibiotics treatment (N; %)** * Mean duration of antibiotics (days + SD) **Steroid treatment (N; %)** * Mean duration of steroids (days; SD) *	23 (6.5) 5.2 + 1.1 5 (1.4) 6.6 + 2.5 0 (0.0) -	63 (63.0) 5.6 + 1.7 14 (14.0) 8.6 + 5.7 11 (11.0) 7.0 + 1.3	<0.001 0.162 <0.001 0.772 <0.001 N/A
**Laboratory investigations** **Complete blood count** • Mean hemoglobin (g/dL + SD) • Mean hematocrit (%+ SD) * • Mean WBC (cells/cumm^3^ + SD) * • Mean ANC (cells/cumm^3^ + SD) * • Mean ALC (cells/cumm^3^ + SD) * • Mean platelets count (cells/cumm^3^ + SD) **Leukocytosis (WBC > 11,000 cells/cumm**^**3**^**) (N;%)** * Lymphopenia (ALC < 1,100 cells/cumm^**3**^**) (N;%)** **Neutropenia (ANC < 1,500 cells/cumm**^**3**^**) (N;%)**	**N = 218 (61.4%)** 12.7 + 1.2 38.6 + 3.4 6327.1 + 2601.1 2446.8 + 1567.6 3120.3 + 1568.1 281755.7 + 97233.0 9 (4.1) 5 (2.3) 65 (29.8)	**N = 64 (64.0%)** 12.3 + 1.7 37.4 + 4.7 8661.1 + 4181.5 3328.6 + 2592.8 4057.7 + 2900.2 305750.0 + 124153.3 15 (23.4) 4 (6.2) 12 (18.7)	0.158 0.047 <0.001 <0.001 0.032 0.050 < 0.001 0.113 0.081

*P < 0.05.

ANC = absolute neutrophil count; ALC = absolute lymphocyte count; BMI = body mass index; SD = standard deviation; WBC = white blood cell; W/H = Weight for height.

Univariate logistic regression revealed that children younger than 1 year old and children with comorbidities were more likely to develop pneumonia with the odds ratios (OR) of 3.10 (95% confidence interval (CI): 1.63–5.90; *p <* 0.001) and 2.43 (95% CI: 1.22–4.84; *p =* 0.009), respectively. Children with pneumonia were more likely to have a leukocytosis with the OR of 7.11 (95% CI: 2.94–17.19; *p <* 0.001). Gender, overweight status (weight for height > + 2 SD), lymphopenia status, and neutropenia status were not associated with children having pneumonia. After adjustment for children younger than 1-year-old and the presence of underlying comorbiditythe multivariate logistic regression still demonstrated that children younger than 1 year old and children with comorbidities were more likely to have pneumonia. The adjusted OR for children younger than 1 year old and children with comorbidities were 2.99 (95% CI: 1.56–5.74; *p =* 0.001) and 2.32 (95% CI: 1.15–4.67; *p =* 0.019), respectively. The data of crude ORs and adjusted ORs for the children with pneumonia were summarized in Table 5.

**Table 5:** Associating factors in children with pneumonia.

	Crude odds ratios (95% CI)	P-value	Adjusted odds ratios (95% CI)	P-value
**Female gender**	1.29 (0.91–1.82)	0.153		
**Age < 1 year**	3.10 (1.63–5.90) *	0.001	2.99 (1.56–5.74) *	0.001
**Underlying disease**	2.43 (1.22–4.84) *	0.009	2.32 (1.15–4.67) *	0.019
**Weight for Height > + 2 SD**	1.05 (0.62–1.77)	0.851		
**WBC count > 11000 cells/cumm** ^**3**^	7.11 (2.94–17.19) *	< 0.001		
**Lymphocyte count < 1100 cells/cumm** ^**3**^ **Neutrophil count < 1500 cells/cumm** ^**3**^	2.84 (0.74–10.91) 0.54 (0.27–1.08)	0.113 0.081		

CI = confidence interval; SD = standard deviation; WBC = white blood cells.

Adjusted for age < 1-year-old and presence of underlying diseases.

*p < 0.05.

## Discussion

To our knowledge, this study was the first epidemiological study of pediatric COVID-19 in Thailand and one of the largest studies in Southeast Asia. With the emergence of the VOC such as the Alpha variant in Thailand in January 2021 and the Delta variant in June 2021, the main area of concern was whether these variants would cause a higher severity in children compared to the wild type of COVID-19. This study revealed that approximately 2.3% of the patients had severe and critical infections and a 0.1% mortality rate. The rate of severe and critical infection in our study was lower than the large epidemiological study by the China CDC which was 5.9% and a systematic review by Cui et al. which was 12% [3,7]. Another study in Italy also found a higher rate of severe disease when compared to our study with the rate of 4.3% [11]. The data in our study was comparable to the study by Hoang et al which revealed 1% of critical infection and 0.1% of mortality rate [6]. Interestingly, our study demonstrated that 12.9% (7/54) of the patients with underlying comorbidities developed severe disease whilst only 1.4% (9/644) of the children without comorbidities would develop severe and critical diseases. This further emphasized the data illustrated by the systematic review by Tsankov et al. that children with comorbidities had a higher risk of developing a more severe course of infection [8].

As the number of infected individuals surged throughout the country, we also observed an increasing number of patients in our care system throughout the study period. During April, the majority of infected individuals were infected with the Alpha variant before the Delta variant took over the majority in late July and August. Despite the small number of critically ill patients in our cohort, we only observed these patients during the last two months of the study, which might correspond to the emergence of the Delta variant in the country. Furthermore, during the two last months of the study, there was a significant decline in patients with asymptomatic infection. Nevertheless, with the data at this moment, it was difficult to extrapolate that the Delta variant was more likely to cause a more severe infection in children compared to the original wild-type variant or the Alpha variant.

Children younger than 1-year-old and children with comorbidities were more likely to develop pneumonia with the adjusted ORs of 2.99 (95% CI: 1.56–5.74; *p =* 0.001) and 2.32 (95% CI: 1.15–4.67; *p =* 0.019), respectively. This coincided with the study by Bellino et al. that children younger than 1-year-old and children with underlying disease were associated with higher severity of the disease [11]. Similarly, upon comparison with a multi-national study in Asia by Wong et al, infant age group and comorbidities were also associated with the more severe clinical course [5]. Unlike the large meta-analysis by Tsankov et al. [8] which stated that obesity was a risk factor for the development of severe disease, overweight and obesity were not associated with the development of pneumonia (at least moderate disease) in our cohort (adjusted OR 1.13 (95% CI: 0.56–2.27; *p =* 0.730)). Children with pneumonia had significantly higher mean WBC, ANC, and ALC counts and were more likely to have leukocytosis compared to the children without pneumonia. These findings were quite similar to the study by Wong et al. that children with severe and critical diseases had significantly higher ANC counts [5]. Due to a limited population being tested for complete blood count in this study, the number of patients with leukocytosis among groups was too few and deemed not adequately powered to perform a multivariate analysis.

The major strengths of this study were that this was the first epidemiological study of pediatric COVID-19 in Thailand and this study was also able to describe the demographic data, clinical characteristics, severity, and outcomes of children in the field hospital and the home isolation program. Unlike other healthcare systems in different countries, we attempted to admit all the patients with positive COVID-19 into the care system and performed regular follow-ups. By setting up the field hospital and the home isolation program, we were able to provide a clearer epidemiological picture of children with COVID-19. Similar to the multi-national study by Wong et al., this study further added that the clinical presentation and severity, as well as the risk factors for the more severe clinical course, were similar in children with COVID-19 throughout Asia.

The major limitation of this study was that this was a single-center study from a tertiary care, university hospital. Thus, this might not reflect the true epidemiological data of the country. Furthermore, the number of patients with severe and critical diseases was small (2.3%), thus there was not enough power to perform the statistical analyses for the risk factors for the development of severe and critical disease. In this study, we tried to compensate the small number of severe and critical populations by using the diagnosis of radiographic pneumonia which was classified as at least moderate severity as a surrogate for more severe disease. However, we still did have a limitation that not everyone in our home isolation cohort received a chest roentgenogram due to transmission concern during transport, which might further limit the power of risk factors determination. Interestingly, we found that 28% of the patients who were diagnosed with pneumonia exhibited no clinical symptoms. These patients were found to have abnormal infiltration on the routine chest x-ray upon admission. Upon follow-up of these patients, none required respiratory support and was discharged clinically well. With our currently limited data, we were unable to make any recommendation for the performance of routine chest x rays in children.

At the time of conducting this study, vaccination in children was still not available and not approved by the local and international Food and Drug Administration. Thus, it was not feasible to outline the effect of vaccination on the severity of children who were vaccinated or not vaccinated. Nevertheless, within this cohort, younger children outside the vaccination age groups were more likely to have a more severe course of the disease. Interestingly, Favipiravir is currently served as a main antiviral agent in both children and adults in our country. The prescription of Favipiravir was based upon the National recommendation guidelines [12]. Due to the limited access and availability of Remdesivir, it was only available to be prescribed for patients with the severe disease after July. This situation is also similar in Turkey, where Favipiravir is recommended in children with severe disease. In this cohort, approximately 16.8% received Favipiravir during treatment ranging from patients with mild disease with comorbidities to patients requiring oxygen supplementation and ventilator support. Our study did not specifically look for the side effects of Favipiravir such as transaminitis, QTc prolongation, or hyperuricemia [13]. Nevertheless, no clinical suggestion of such conditions was reported in our cohort; thus, we did not perform any laboratory follow-up in patients who received Favipiravir. The majority of febrile patients had a subsiding fever within 48 hours. This was also demonstrated in the study by Ozsurekci et al. that Favipiravir was well tolerated without major side effects in children with renal impairment [14]. Since our study was a retrospective descriptive study, it was not feasible to determine the effectiveness of Favipiravir from the current data.

Despite our limitations, we still demonstrated similar risk factors as compared to other previous studies. A larger, multi-center study or a national registry study would be warranted to determine the risk factors for the development of a more severe course of the disease.

## Conclusion

In the era of the variants of concern, the proportion of children with severe and critical diseases remained low. However, prudence must be taken in caring for younger children and children with comorbidity.
